# Detection and characterization of *Plasmodium* spp. by semi-nested multiplex PCR both in mosquito vectors and in humans residing in historically endemic areas of Paraguay

**DOI:** 10.1016/j.parepi.2020.e00174

**Published:** 2020-08-15

**Authors:** Florencia del Puerto, Mónica Ozorio, Beatriz Trinidad, Nidia Martínez, Martha Torales, Luciano Franco, Luis Ferreira, Ninfa Vera de Bilbao

**Affiliations:** a National University of Asunción, Research Institute in Health Science (IICS-UNA), Department of Tropical Medicine, San Lorenzo, Paraguay; b National Program of Malaria Control, National Service of Malaria Elimination (SENEPA), Ministry of Public Health and Social Welfare, Asunción, Paraguay; c Department of Entomology, National Service of Malaria Elimination (SENEPA), Ministry of Public Health and Social Welfare, Asunción, Paraguay; d Technical Management Office, National Service of Malaria Elimination (SENEPA), Ministry of Public Health and Social Welfare, Asunción, Paraguay

**Keywords:** Malaria, PCR, Paraguay

## Abstract

In Paraguay, no cases of Malaria have been recorded since 2011. Microscopy and the SnM-PCR technique were implemented to detect and characterize *Plasmodium* spp. both in mosquitoes and in humans residing in historically endemic areas of Paraguay, to evaluate the possibility of finding asymptomatic cases and/or Plasmodium parasites circulating in anophelines. Between 2013 and 2015, 361 human blood samples were collected on filter paper, and between 2016 and 2017, 938 female *Anopheles* mosquitoes were captured in 15 Paraguayan localities. All the diagnostic techniques showed negative results. We observed no asymptomatic case or *Plasmodium* circulating in vectors.

In Latin America, between 2000 and 2011, 13 out of the 21 countries endemic for malaria managed to reduce the number of cases (confirmed by microscopy) and the ranges of incidence cases by more than 75% (Ministerio de Salud Pública y Bienestar Social (MSPyBS), 2011; World Health Organization (WHO) World Malaria Report, 2012). In particular, Paraguay was able to reduce the number of cases by 97%.

In Paraguay, between 2001 and 2011, malaria cases came only from the three most endemic departments: Alto Paraná, Caaguazú and Canindeyú (Organización Panamericana de la Salud (OPS), 2018). It is also important to point out that, in 2012, Paraguay was among the six countries that were at the malaria pre-elimination stage (Ministerio de Salud Pública y Bienestar Social (MSPyBS), 2011), which led the country to earn the first place in the contest “Champions Against Malaria 2012”, awarded by the Pan-American Health Organization (PAHO), because no cases had been reported since 2011 (Organización Panamericana de la Salud /Organización Mundial de la Salud (OPS/OMS), 2012).

In Paraguay, only *Plasmodium vivax* is indigenous, and the cases of *Plasmodium falciparum* reported in recent years have been of people who had imported the infection from other countries, mainly African countries. In countries of low and moderate endemicity, the high prevalence of asymptomatic cases with *P. vivax* reported by the World Health Organization (WHO) and the operational difficulty of radical treatment of hypnozoites (the latent form of *P. vivax*) suggest that the probability of eliminating *P. vivax* is lower than that of eliminating *P. falciparum* (World Health Organization (WHO), 2007; Wells, 2010; Waltmann et al., 2015).

In tropical areas, the latency period of hypnozoites is shorter, whereas the relapse time may vary. In particular, *P. vivax* appears to have a longer latency in the Americas than in Asia and the Pacific (World Health Organizatio (WHO), 2015). In Paraguay, asymptomatic cases with *P. vivax* were detected by the National Service for the Eradication of Malaria (Servicio Nacional de Erradicación del Paludismo; SENEPA) in 1999, after detecting 9946 febrile and non-febrile cases (Wells, 2010).

The gold standard diagnostic method for malaria is microscopy (Wongsrichanalai et al., 2007). In contrast, the seminested multiplex PCR (SnM-PCR) technique has been shown to be more sensitive and specific for the detection of *Plasmodium* parasites than microscopy, detecting 12.4% more of positive samples and 13% of undetectable infections by Giemsa staining (Rubio et al., 1999; Snounou et al., 1993).

Based on the above, the specific aim of this study was to implement SnM-PCR to detect and characterize *Plasmodium* in blood samples from humans residing in historically endemic areas of Paraguay to verify the possibility of finding asymptomatic cases, as well as in samples from *Anopheles* vectors, to determine the possibility of finding circulating *Plasmodium* parasites (Fig. 1).

**Fig. 1:**
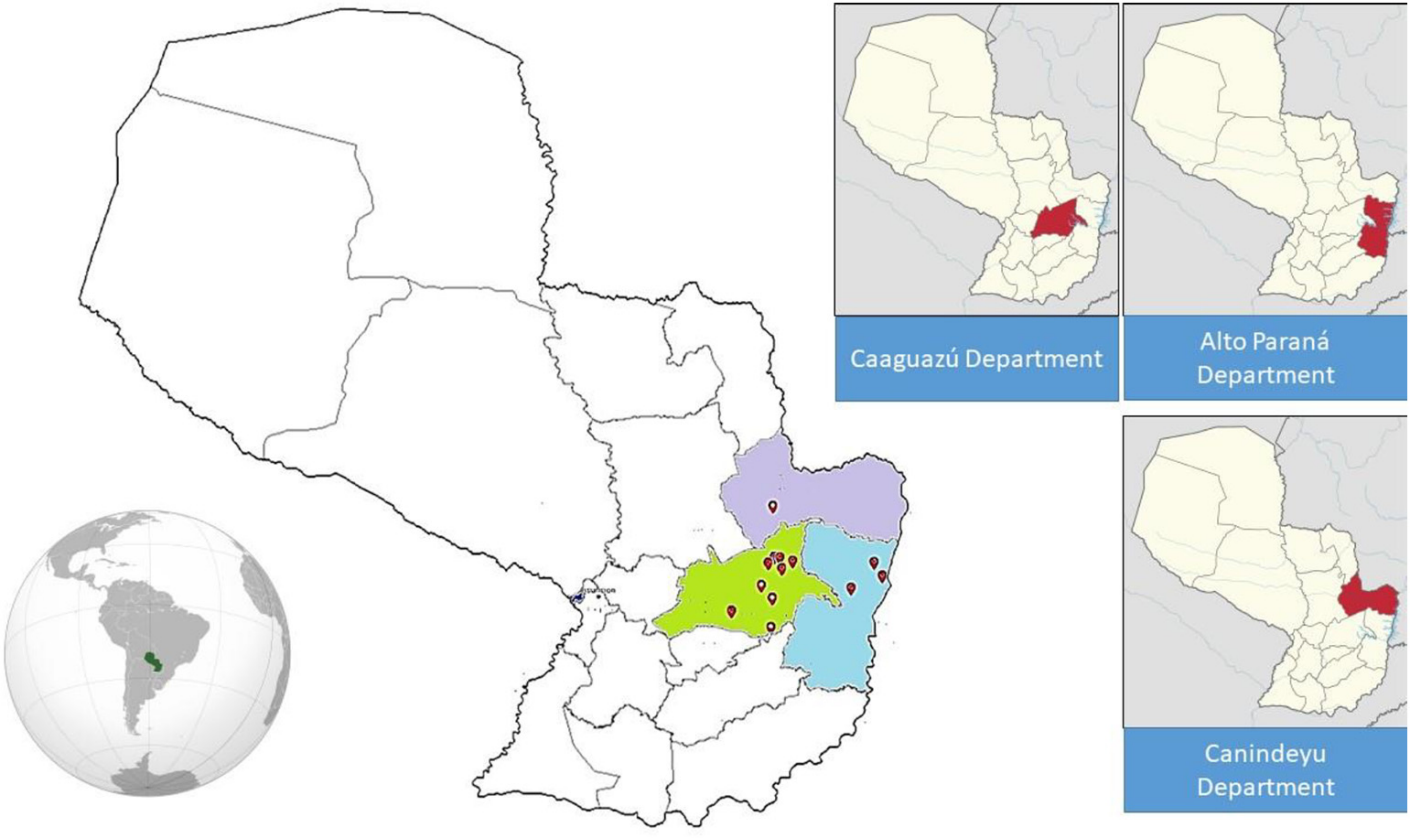
Map of Paraguay where the collect of human samples and mosquitoes were performed.

The study was a descriptive cross-sectional study. The human blood samples were collected between September 2013 and May 2015, and female mosquitoes of the genus *Anopheles* were captured between 2016 and 2017. The human sample size was calculated taking into account the record of cases reported to the National Program of Malaria Control of the SENEPA between 2007 and 2011 (i.e. the year in which the last case of malaria was recorded) and the localities were chosen based on where those cases came from.

For molecular analysis by SnM-PCR, the blood samples were placed on filter paper 903.

For microscopic analysis, blood samples were taken from the same puncture for both thick blood drop and smears.

With the collaboration of the SENEPA, *Anopheles* mosquitoes were captured from natural breeding sites in the above-mentioned localities during August, September and November 2016 and December 2017. In Paraguay, these months correspond to austral spring and are characterized by warm temperatures.

The *Anopheles* females were identified using classification keys (Gorham et al., 1967; Consoli and de Oliveira, 1994), selected, and then deposited in 1.5-mL propylene tubes. Pools of a maximum of 10 female mosquitoes as Rondon et al (Rondón et al., 2019) per species per locality were transported to the Instituto de Investigaciones en Ciencias de la Salud (IICS-UNA), where they were stored at −20°C until studied.

The filter papers were cut by delimiting the area of the drop of blood, and the blood samples were then subjected to DNA extraction by using the Chelex method described by Rubioet al (Rubio et al., 1999).

The mosquito pools were subjected to DNA extraction by using the NucleoSpin® DNA Insect Kit from Macherey-Nagel GmbH & Co., Düren, Germany, following the manufacturer's protocol.

For the identification of Plasmodium spp. in human blood samples, the amplification protocol, the concentrations, the primer sequences and the size of the fragments were the same as those published by Ta Tang et al (Ta Tang et al., 2011). Briefly, this technique is a Semi-Nested Multiplex Malaria PCR, consisting of two multiple PCR processes (PCR1 and PCR2). DNA of four human infecting plasmodiums can be detected by the amplified fragment size characteristic of each of them. The DNA target is the small subunit of the 18S RNA (ssrDNA) that is even used to amplify human DNA as internal control in order to avoid false negatives.

The master mix for 50 ul of reaction in PCR1 contain: 1× Buffer, 2 mM MgCl_2_, 0.2 mM dNTPs, 0.1 uM primer PLF (5´-AGTGTGTATCAATCGAGTTTC-3′), 0.1 uM primer REV (5´-GACGGTATCTGATCGTCTTC-3′), 0,0125 uM primer HUF (5´-GAGCCGCCTGGATACCGC-3′), 1,25 U Taq DNA polymerase instead of Tth polymerase as it is mentioned in the protocol and 5 ul of DNA. The master mix for 25 ul reaction in PCR2 contain: 1× Buffer, 2 mM MgCl2, 0.2 mM dNTPs, 0.15 uM primer NEWPLFSHORT (5′-CTATCAGCTTTTGATGTTAG-3′), 0.10 uM primer MALSHORT (5´-TCCAATTGCCTTCTG-3′), 0.15uM primer FALSHORT (5′- GTTCCCCTAGAATAGTTACA-3′), 0.10 uM primer OVASHORT (5´-AGGAATGCAAAGARCAG-3′), 0.10 uM primer VIVSHORT (5´-AAGGACTTCCAAGCC-3′), 0.7 U Taq DNA polymerase instead of Tth polymerase as it is mentioned in the protocol and 2 ul amplified DNA at PCR1.

The cycling for PCR1 include an initial denaturation for 5 min at 94 °C, 40 cycles of 20 s at 94 °C, 20 s at 62 °C and 30 s at 72 °C, and a final elongation for 10 min at 72 °C.

The amplified products at PCR1 are: 783 bp* (*P. malariae, P. falciparum, P. ovale, P. vivax*) and 231 bp for human internal control, respectively. *Only visualized in the gel in those parasitaemia cases with levels higher than 3%.

The amplified products at PCR2 are: 241 bp (*P. malariae*), 370 bp (*P. falciparum*), 407 bp (*P. ovale*), 476 bp (*P. vivax*) respectively.

For the identification of Plasmodium spp. in mosquitoes' samples, the amplification protocols were the same as those published by Ta Tang et al (Ta Tang et al., 2011) with a small modification that consisted in designing and incorporating a pair of primers that allowed the amplification of *Anopheles* DNA for the same purpose as human DNA as a positive internal control. The modification (forward 5 ´ CTS AAT TAG GWC ATC CWG GA 3 ´ and reverse 5 ´ CAT GAG CAA TTC CWG ATG AWA GA 3 ´). This gave as a product a 300-bp fragment, different from the sizes of the expected fragments for *P. malariae* (241 bp), *P. falciparum* (370 bp), *P. ovale* (407 bp) and *P. vivax* (476 bp) and we incorporated the primers for *Anopheles* in both steps (PCR1 and PCR2) and the product was observed when running the gel of PCR2.

For the design of the primers, we aligned sequences of the subunit of the gene of cytochrome *c* oxidase 1 (COX1) (GenBank access numbers: KP193458, KC330237, GU226668, KJ492419, JF923728, JF923727, JF923729, JF923701 and JN413689). Positive controls for the PCR reactions were amplified from the genomic DNA of *P. falciparum* RO-33, MRA-200G, obtained through the MR4 as part of the BEI Resources Repository, NIAID, NIH.

The sensitivity test was done by 1/10 serial dilution of *P. falciparum* genomic DNA starting from 10 ng. A total of 7 duplicate samples were prepared with 25 ul of human blood and we have observed parasite at a concentration of up to 0.01 ng, a fact mentioned in the present manuscript.

The PCR products were analyzed through electrophoretic runs in 2% agarose gels, using TAE 1× buffer. The sizes of the bands were verified by running in parallel a 100-bp marker and visualized with UV light after staining with ethidium bromide. Pictures of the gels were taken for their documentation.

The blood samples for microscopy were stained with Giemsa staining plus buffer solution, and then fixed with absolute methanol. The samples were observed with a 100× oil immersion objective. A preparation was considered negative once 500 fields of the thick drop and 800 fields of the thin smear had been carefully examined.

All the selected individuals were over 18 years of age who agreed to participate.

The data were handled as confidential and the names of the individuals were codified for the management of the samples in the laboratory.

This project was approved by the Ethics and Scientific Committees of the IICS-UNA under code P08/2013.

Table 1 shows the molecular analysis of the human blood samples obtained in filter paper, and the mosquitoes captured. None of the human samples collected in the 15 localities showed circulating *Plasmodium* parasites either by microscopy (the gold standard method) or by the SnM-PCR technique.

**Table 1:** Detection of *Plasmodium* spp. by SnM-PCR and microscopy in human blood samples and Anopheles mosquitoes.

			SnM-PCR		
Department	Localities	Human samples analyzed (*N* = 361)	*Plasmodium spp*	Internal positive control	Microscopy
Alto Paraná	Misión Verbo Divino	39	–	+	–
Alto Paraná	Mbaracamoa	35	–	+	–
Alto Paraná	Nueva Esperanza	26	–	+	–
Canindeyú	Pira Vera	38	–	+	–
Caaguazú	Mbarigui	6	–	+	–
Caaguazú	San Juan Indígena	8	–	+	–
Caaguazú	Nueva Esperanza	13	–	+	–
Caaguazú	Palmares Mil Palos	57	–	+	–
Caaguazú	Yby Moroti	18	–	+	–
Caaguazú	Cnel. Toledo	66	–	+	–
Caaguazú	Santa Clara	8	–	+	–
Caaguazú	Santa Teresa	25	–	+	–
Caaguazú	Ñu Jhovy	7	–	+	–
Caaguazú	Pindo'í	12	–	+	–
Caaguazú	Nueva Brasilia	3	–	+	–


			SnM-PCR	
Species		N captured/N pools	*Plasmodium spp*	Positive pools for internal positive control N(%)
*Anopheles albitarsis*		558/62	–	61(98.3)
*Anopheles evansae*		60/7	–	4(57.1)
*Anopheles fluminensis*		9/4	–	1(25.0)
*Anopheles galvaoi*		7/2	–	2(100.0)
*Anopheles noroestensis*		8/2	–	2(100.0)
*Anopheles nuneztovari*		3/1	–	1(100.0)
*Anopheles oswaldoi*		19/3	–	2(66.7)
*Nyssorynchela parvus*		14/6	–	0(0.0)
*Anopheles rondoni*		5/3	–	3(100.0)
*Anopheles shanonni*		2/1	–	0(0.0)
*Anopheles strodei*		202/26	–	22(84.6)
*Anopheles triannulatus*		41/5	–	2(40.0)
*Albitarsis complex*		1/1	–	1(100.0)
*Nyssorhynchela lutzi*		9/2	–	2(100.0)

Table 2 shows the number and species of *Anopheles* female mosquitoes captured by locality. *A. albitarsis* was the most prevalent (59.4%), followed by *A. strodei* (21.5%), whereas *A. darlingi* was absent in all the localities surveyed. Curiously, most of the species found in less frequency were captured in habitats where the presence of *A. albitarsis* was null or low, demonstrating some kind of competition among them.

**Table 2:** Number and species of *Anopheles* female mosquitoes captured by locality.

Departments	Species → Locality ↓	Anopheles albitarsis	Anopheles evansae	Anopheles fluminensis	Anopheles galvaoi	Anopheles noroestensis	Anopheles nuneztovari	Anopheles oswaldoi	Nyssorynchela parvus	Anopheles rondoni	Anopheles shanonni	Anopheles strodei	Anopheles triannulatus	Albitarsis complex	Nyssorhynchela lutzi	Total
Alto Paraná	**Nueva Esperanza**	119								3		6				**128**
	**Misión Verbo Divino**	1	9					1				6				**17**
	**Maracamoa**	3	45	2	6	7		18	1			46	40			**168**

Canindeyù	**Pira Vera**															**0**
Caaguazú	**Santa Teresa**	310								1	2	1				**314**
	**Coronel Toledo**	4														**4**
	**Nueva Brasilia**			2								20	1			**23**
	**Santa Clara**	102														**102**
	**Mbarigui**	1	6									9		1	1	**18**
	**San Juan Indígena**	8		1					10			101			8	**128**
	**Nueva Esperanza**	5							3			5				**13**
	**Pindo'í**	2		4		1	3					7				**17**
	**Mil Palos**	3														**3**
	**Yvy Moroti**				1					1		1				**3**
	**Ñu Jhovy**															**0**

	**Total**	**558**	**60**	**9**	**7**	**8**	**3**	**19**	**14**	**5**	**2**	**202**	**41**	**1**	**9**	**938**
	**(%)**	59.5	6.4	1.0	0.7	0.9	0.3	2.0	1.5	0.5	0.2	21.5	4.4	0.1	1.0	**100**

The primers designed showed high specificity for the mosquito species collected, primarily for *A. albitarsis* and *A. strodei*, which were considered secondary vectors in the local transmission of malaria in the country (Table 2 and Fig. 2).

**Fig. 2:**
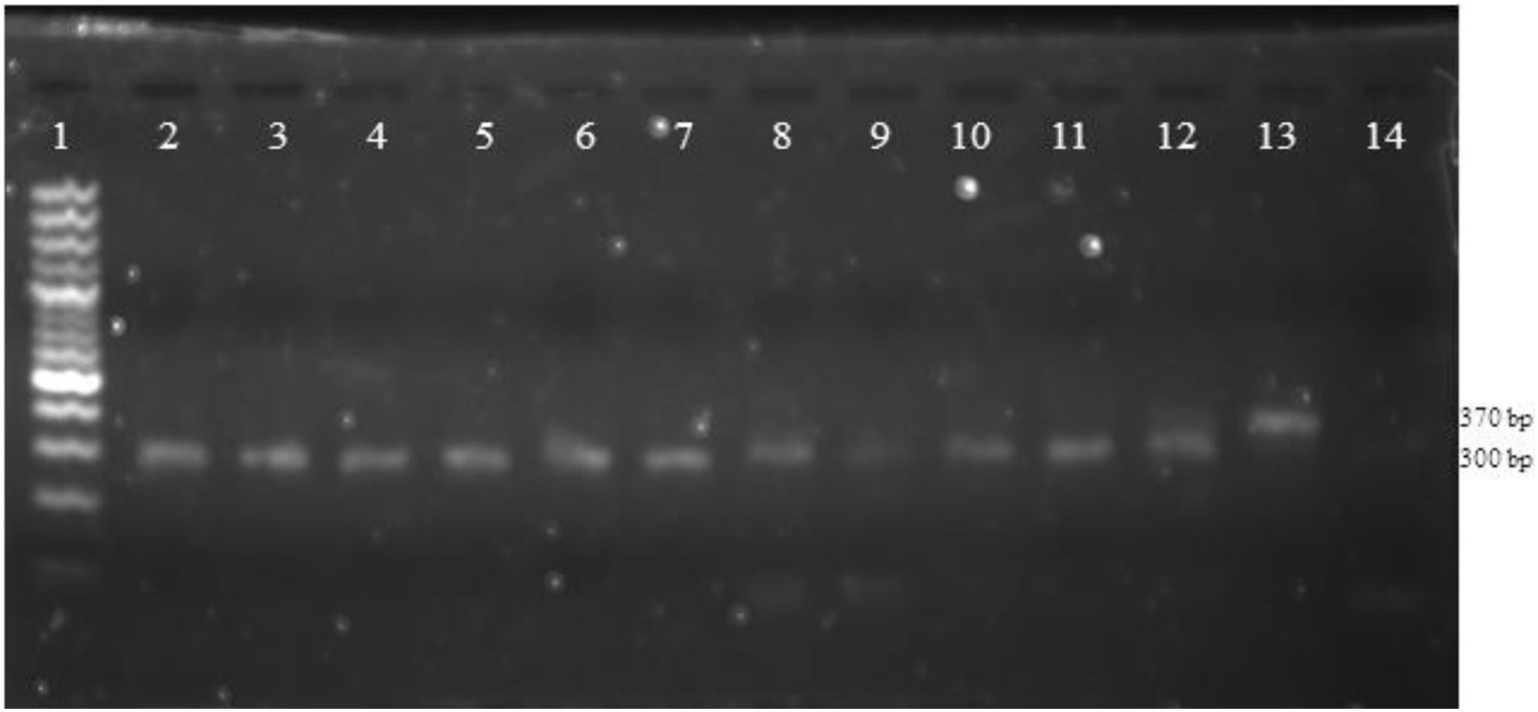
Electrophoretic run where the amplified internal positive control fragment is observed for the species of *Anopheles* mosquitoes analyzed. PCR2. 1: 100-bp marker, 2: *Anopheles albitarsis*, 3: *Anopheles. strodei*, 4: *Anopheles galvaoi*, 5: A*nopheles. oswaldoi*, 6: *Anopheles nunestovari*, 7: *Anopheles fluminensis*, 8: *Anopheles triannulatus*, 9: *Anopheles rondoni*, 10: *Anopheles evansae*, 11: *Anopheles noroestensis*, 12: *Nyssorhynchela lutzi*, 13: positive control: *Plasmodium falciparum*, 14: negative control (PCR mix).

At the time this study was conducted, the country was already at the malaria elimination phase. Indeed, the results of this study have been recently taken into account by foreign experts who assessed the country situation to grant Paraguay the certification of malaria-free country in June 2018. One of the main objectives of this assessment was to verify the absence of local transmission of malaria in historically endemic regions.

Because of the historical behavior of the transmission of the disease, all the eastern region of Paraguay presents conditions for the development of malaria-transmitting vectors. However, in this region, the Departments with higher risk of re-establishment of the disease are the Departments of Alto Paraná, Caaguazú and Canindeyú (World Health Organization (WHO). World Malaria Report, 2010; Servicio Nacional de Erradicación del Paludismo (SENEPA), 2017) Although the native malaria parasite in Paraguay is *P. vivax*, which is characterized by being able to remain in a state of latency in the form of hypnozoites, failure to observe them in circulation in asymptomatic way in any of the individuals (considering the size of the sample) is a positive and encouraging result.

In terms of vectors, although no circulating *Plasmodium* parasites were detected, it is quite interesting to analyze their ecology (Table 2). Firstly, it is important to note that the Field Manual for Entomological Surveillance of *Anopheles* of the SENEPA(Departamento de Entomología del Servicio Nacional de Erradicación del Paludismo (SENEPA), 2013) makes a historical overview of the presence of anopheline vectors in Paraguay, which establishes the presence of *A. darlingi* since 1951 and the predominance of *A. albitarsis*. It is interesting to follow the timeline described in this Manual because it makes reference to the period between 1967 and 1968, when, despite identifying 31 anopheline species, both vectors mentioned above (*A. darlingi* and *A. albitarsis*) remained being the most frequent, as well as to the period between 1992 and 1993, when it was noted that 20% of the vectors captured corresponded to *A. darlingi*, considered the main vector at the local level (Departamento de Entomología del Servicio Nacional de Erradicación del Paludismo (SENEPA), 2013).

Interestingly, in the present study, performed in 2016 and 2017, no *A. darlingi* individuals were found in any of the localities surveyed, being *A. albitarsis* the most prevalent (59.4%), followed by *A. strodei* (21.5%).

The data mentioned in the Field Manual for Entomological Surveillance of *Anopheles* of the SENEPA(Departamento de Entomología del Servicio Nacional de Erradicación del Paludismo (SENEPA), 2013) correspond to captures performed at the banks of the Paraná River and the banks of the Itaipú Lake, on the Paraguayan side. Most of the localities sampled in this study had the same forest characteristics, which, until today, are only seen near the Itaipú Lake, an area considered a natural reserve. However, nowadays, these localities are practically surrounded by large soybean crops, where forests are null or insufficient. The massive agricultural exploitation of the land has also directly contributed to the disappearance of habitats of these species and is probably one of the strongest reasons that may explain the absence of *A. darlingi*, which, as mentioned above, is considered the main vector of malaria at the national level.

The density of these *Anopheles* species is also being affected by the environmental impact mentioned above. This constitutes an important factor for the decrease of the risk of vector-borne transmission in many of these localities, except for Nueva Esperanza in Alto Paraná Department and Santa Teresa and Santa Clara in Caaguazú Department, where there is still a high density of *A. albitarsis*. In addition, these localities are surrounded by rivers like the Yguazú River, lakes like the Yguazú Lake, and marsh areas, which make it possible to maintain natural breeding sites for these mosquitoes.

Regarding the molecular technique applied to the mosquito samples, we designed a pair of primers for the SnM-PCR that can act as positive controls in the detection of *Plasmodium* spp. in *Anopheles* female mosquitoes. Our results showed high specificity for the species collected, primarily for *A. albitarsis* and *A. strodei*, which were considered secondary vectors in the local transmission of Malaria (Fig. 2).

In the other species captured in lower number, the percentage of negative results varied, which seems to indicate either that these primers are not specific (as seems to be the case for *A*. (*Nys*) *parvus* and *A. shanoni*) or that it is a problem of sensitivity probably due to a very low performance of the DNA extraction (0.6–3 ng/μL). This should be elucidated by increasing the number of samples to reach a conclusion about the sensitivity and specificity of this pair of primers for these species.

## Financial support

Consejo Nacional de Ciencia y Tecnología (CONACYT), Fondo para la Excelencia de la Educación y la Investigación (FEEI), Paraguay. Project number 14-INV-463.

## Author's contribution

**Florencia del Puerto**: wrote the protocol, obtained funds, performed the molecular.

techniques and wrote the manuscript.

**Monica Ozorio:** performed the microscopic analysis.

**Beatriz Trinidad:** performed the microscopic analysis.

**Nidia Martínez**: taxonomic identification of the mosquitoes.

**Martha Torales**: coordinated the field trips for biological samples collection.

**Luciano Franco**: taxonomic identification of the mosquitoes.

**Luis Ferreira**: taxonomic identification of the mosquitoes.

**Ninfa Vera de Bilbao**: analysis of the results.

## Declaration of Competing Interest

Detection and characterization of *Plasmodium* spp. by semi-nested multiplex PCR both in mosquito vectors and in humans residing in historically endemic areas of Paraguay.

The authors declare that there is no conflict of interest.
